# Dataset from 55 experts engaged in nature conservation in Mozambique

**DOI:** 10.1016/j.dib.2019.105080

**Published:** 2020-01-03

**Authors:** Aires Afonso Mbanze, Natasha Sofia Ribeiro, Carina Vieira da Silva, José Lima Santos

**Affiliations:** a Faculty of Agrarian Sciences, Universidade Lúrio, Department of Environment and Nature Conservation, Niassa Province, Campus Universitários de Unango, Sanga District, Mozambique; b Nova School of Business and Economics, Universidade Nova de Lisboa, Campus de Carcavelos, Rua da Holanda, P.O. Box., 2775-405, Lisbon, Portugal; c Centre for Forest Studies (CEF), Instituto Superior de Agronomia (ISA), Universidade de Lisboa, Tapada da Ajuda, P.O. Box, 1349-017, Lisbon, Portugal; d Eduardo Mondlane University, Faculty of Agronomy and Forest Engineering, Av. J. Nyerere 3453/Campus Universitário Principal, Maputo, Mozambique; e MARE - Marine and Environmental Sciences Centre, Faculdade de Ciências, Universidade de Lisboa, Av. Nossa Sra do Cabo 939, 2750-374, Cascais, Portugal

**Keywords:** Conservation experts, Developing countries, Perceived views and Niassa National Reserve

## Abstract

The data of this article is related to the original article entitled *“An expert-based approach to assess the potential for local people engagement in nature conservation: The case study of the Niassa National Reserve in Mozambique”* [1]*,* published in *Journal for Nature Conservation*. The dataset is from an online and self-administrated survey with 55 experts aware of conservation policies and incentives under implementation in the Niassa National Reserve (NNR), the largest protected area in the country and third-largest in Africa. The survey included four sections of both compulsory and non-compulsory questions, mostly in closed-ended Likert-scale. In the first section, experts were asked about the main practices that threaten biodiversity conservation in the NNR, the actors who are directly and indirectly responsible for each practice, and the reasons for local people's involvement with those practices. The second section was about the effectiveness and limitations of the current compensation measures to engage local residents with conservation-friendly practices. In the third section, respondents were asked to select new measures to enhance the current conservation status and engage local people more effectively in conservation. The last section was about the socio-economic profile of respondents. The survey was conducted from June to September 2017. The paper includes the survey itself, raw data in an Excel spreadsheet, descriptive analysis, crosstabulation and Post Hoc cellwise tests (goodness of fit). Data are provided for public use and can serve as a benchmark for collaboration in order to conduct more comprehensive research, comparative analysis as well as panel data can be derived. This data can also have applications in other fields such as mathematics, statistics, and computation.

**Table:** Specifications Table

Subject area	Environmental science
More specific subject area	Management, Monitoring, Policy, and Law
Type of data	*Excel files, table and online questionnaires*
How data was acquired	*Online* and self-administration survey
Data format	*Raw, filtered and analysed*
Experimental factors	*Respondents were selected based on education, number of visits, time spent while visiting, the objective of the visit and years of experience in conservation*
Experimental features	*Online* and self-administration survey was conducted to 55 experts engaged in conservation in Mozambique, from June to September 2017
Data source location	*Mozambique countrywide (mainly in Maputo city, Lichinga city, Mecula, Marrupa and Mavago districts in the Niassa Province, closer to the Niassa National Reserve)*
Data accessibility	*Data are available with this article*
Related research article	Author's name: Aires Afonso Mbanze, Natasha Sofia Ribeiro, Carina Vieira da Silva and José Lima Santos Title: *“An expert-based approach to assess the potential for local people engagement in nature conservation: The case study of the Niassa National Reserve in Mozambique”* Journal: Journal for Nature Conservation DOI: https://doi.org/10.1016/j.jnc.2019.125759

**Table:** 

**Value of the data** • Data can be used for site comparison among different conservation areas; • Data can serve as a benchmark for further collaborative research; • The questionnaire can be replicable and improved in future studies; • Data can be analysed on different ways to come up with other possible scenarios to advise decision-makers and conservation experts on how to improve conservation of protected areas in developing countries; • Data can also be used in other fields, including statistics and computer sciences.

## 1 Data

The dataset of this article is related to experts' views about conservation policies and incentives implemented at Niassa National Reserve (NNR). The questionnaire used to generate the dataset is presented in Appendix A. Raw Excel dataset is online available on mendely data (https://data.mendeley.com/datasets). The detailed information regarding the profile of respondents is presented in Table 1. Table 2 presents more detailed information about the socio-demographic information of respondents. Table 3 presents the different rating scales used for each major themes; Table 4, Table 5, Table 6, Table 7, Table 8 are the post-hoc cellwise comparisons between major themes with meaningful explanation; and Table 9 presents a post-hoc cellwise test between experts’ level of education and the level of improvement of different attributes after implementation of new proposed measures.

**Table 1:** Organizations from which the surveyed respondents were selected.

Organization	Number of respondents (%)
Conservation NGOs	9 (16)
Private sector (concessionaries of Hunting Blocks)	4 (7)
**Governmental institution**	
National Ministry of Land, Environment and Development	5 (9)
Provincial and district environment and conservation related institution	19 (35)
**Academic Institutions**	
Universities and Technical Institutes	10 (18)
Research institutions	2 (4)
Others	6 (11)
**Total**	55 (100)

**Table 2:** Socio-demographic information of respondents.

N^o^	Variables	Frequency	Percentage (%)
1	**Gender**		
	Male	43	78.2
	Female	12	21.8
2	**Education**		
	Professional Education (basic or secondary)	15	27.3
	Upper Secondary School	6	10.9
	Higher Education	34	61.8
3	**Major Field**		
	Agriculture	32	58.2
	Biology	4	7.3
	Social Sciences	9	16.4
	Others	10	18.2
4	**How long have you stayed there?**		
	Any time	12	21.8
	less than a month	13	23.64
	1–4 Months	10	18.2
	5–8 months	2	3.6
	8–12 months	3	5.5
	>12	15	27.3
5	**The main objective of your trip**		
	Working	29	52.7
	Research	11	20
	Just passing through	1	1.8
	Tourism	4	7.3
	Visit	1	1.8
	Others	9	16.4
6	**Years of experience in conservation**		
	1–2	16	31.37
	3–5	19	37.25
	6–10	12	23.53
	>10	4	7.84

**Table 3:** Rating scale coded for the four major themes that experts were requested to answer to.

Nº	Major themes	Rating scale	Source
Q.1	Identify the degree of threat each of the existing problems in the NNR represents for conservation	0 = very little, 1 = little, 2 = moderate, 3 = high and 4 = very high	[[2], [3], [4], [5], [6], [7], [8], [9]]
Q.1.1	Among different actors, indicate the main responsible for each of these threats.	0 = No, 1 = Yes	
Q.2	Several reasons for local people to be involved with practices that threaten conservation	2 = strongly agree, 1 = agree, 0 = undecided, −1 = disagree and −2 = strongly disagree	[2,8,[10], [11], [12], [13]]
Q.3	Put the current compensation measures in order of importance to the local population	6 = most important to 1 = least important	
Q.3.1	Limitations with the way that current compensation measures are being delivered	2 = strongly agree, 1 = agree, 0 = undecided, −1 = disagree and −2 = strongly disagree	[2,7,14]
Q.4	What will be the effectiveness of each new measures below in order to promote the adoption of conservation-friendly practices	2 = very positive, 1 = positive, 0 = no effect; −1 = negative and −2 = very negative	[2]
Q.4.1	Level of improvement with adoption of new measures	4 = 76–100%, 3 = 51–75%, 2 = 26–50%, 1 = 1–25% and 0 = 0%	Authors
Q.4.2	Level of improvement in people behaviours and motivation for conservation	4 = very high, 3 = high, 2 = Moderate, 2 = low and 0 = Null

**Table 4:** Post-hoc cellwise tests between clusters of the degree of threat that each of the existing problems in the NNR represents (Q.1), and reasons for local people engagement in threatening practices (Q.2).

			Q.1		
			N1	N2	N3
Q.2	N1	Count	7	0	2
		Expected Count	4.3	2.5	2.3
		% within Ward Method	77.8%	0.0%	22.2%
		Adjusted Residual	2.0	−2.0	−0.2
		P (Z_ij_)	0.0450	0.0446	0.8077
	N2	Count	0	5	3
		Expected Count	3.8	2.2	2.0
		% within Ward Method	0.0%	62.5%	37.5%
		Adjusted Residual	−2.9	2.4	0.8
		P (Z_ij_)	0.0038	0.0155	0.3975
	N3	Count	12	8	6
		Expected Count	12.3	7.1	6.6
		% within Ward Method	46.2%	30.8%	23.1%
		Adjusted Residual	−0.2	0.6	−0.4
		P (Z_ij_)	0.8750	0.5814	0.7015
	N4	Count	1	1	2
		Expected Count	1.9	1.1	1.0
		% within Ward Method	25.0%	25.0%	50.0%
		Adjusted Residual	−0.9	−0.1	1.2
		P (Z_ij_)	0.3542	0.9156	0.2419
	N5	Count	6	1	1
		Expected Count	3.8	2.2	2.0
		% within Ward Method	75.0%	12.5%	12.5%
		Adjusted Residual	1.7	−1.0	−0.9
		P (Z_ij_)	0.0893	0.3102	0.3629

**Table 5:** Post-hoc cellwise tests between clusters of the degree of threat that each of the existing problems in the NNR represents (Q.1) and compensation measures currently in place at the reserve (Q.3).

			Q.1		
			N1	N2	N3
Q.3	N1	Count	26	0	0
		Expected Count	12.3	7.1	6.6
		% within Ward Method	100.0%	0.0%	0.0%
		Adjusted Residual	7.4	−4.3	−4.1
		P (Zij)	0.0000	0.0000	0.0000
	N2	Count	0	15	0
		Expected Count	7.1	4.1	3.8
		% within Ward Method	0.0%	100.0%	0.0%
		Adjusted Residual	−4.3	7.4	−2.7
		P (Zij)	0.0000	0.0000	0.0080
	N3	Count	0	0	14
		Expected Count	6.6	3.8	3.6
		% within Ward Method	0.0%	0.0%	100.0%
		Adjusted Residual	−4.1	−2.7	7.4
		P (Zij)	0.0000	0.0080	0.0000

**Table 6:** Post-hoc cellwise tests between clusters of the degree of threat that each of the existing problems in the NNR represents for conservation and level of improvement of different ecosystem services, after the implementation of new measures.

			Q.4.1		
			C1	C2	C3
Q.1	C1	Count	20	0	0
		Expected Count	9.5	5.5	5.1
		% within Ward Method	100.0%	0.0%	0.0%
		Adjusted Residual	5.9	−3.4	−3.3
		P (Zij)	0.0000	0.0006	0.0011
	C2	Count	0	8	5
		Expected Count	6.1	3.5	3.3
		% within Ward Method	0.0%	61.5%	38.5%
		Adjusted Residual	−3.9	3.2	1.2
		P (Zij)	0.0001	0.0015	0.2179
	C3	Count	6	7	9
		Expected Count	10.4	6.0	5.6
		% within Ward Method	27.3%	31.8%	40.9%
		Adjusted Residual	−2.4	0.6	2.1
		P (Zij)	0.0153	0.5366	0.0317

**Table 7:** Post-hoc cellwise tests between clusters of reasons for local people being involved with practices that threaten conservation, (Q.2) and compensation measures currently in place at the reserve (Q.3).

			Q.3		
			N1	N2	N3
Q.2	N1	Count	7	0	2
		Expected Count	4.3	2.5	2.3
		% within Ward Method	77.8%	0.0%	22.2%
		Adjusted Residual	2.0	−2.0	−0.2
		P (Zij)	0.0450	0.0446	0.8077
	N2	Count	0	5	3
		Expected Count	3.8	2.2	2.0
		% within Ward Method	0.0%	62.5%	37.5%
		Adjusted Residual	−2.9	2.4	0.8
		P (Zij)	0.0038	0.0155	0.3975
	N3	Count	12	8	6
		Expected Count	12.3	7.1	6.6
		% within Ward Method	46.2%	30.8%	23.1%
		Adjusted Residual	−0.2	0.6	−0.4
		P (Zij)	0.8750	0.5814	0.7015
	N4	Count	1	1	2
		Expected Count	1.9	1.1	1.0
		% within Ward Method	25.0%	25.0%	50.0%
		Adjusted Residual	−0.9	−0.1	1.2
		P (Zij)	0.3542	0.9156	0.2419
	N5	Count	6	1	1
		Expected Count	3.8	2.2	2.0
		% within Ward Method	75.0%	12.5%	12.5%
		Adjusted Residual	1.7	−1.0	−0.9
		P (Zij)	0.0893	0.3102	0.3629

**Table 8:** Post-hoc cellwise tests between compensation measures that are currently in place at the reserve (Q.3) and level of improvement of different ecosystem services, after the implementation of new measures (Q.4.1).

			Q.4.1		
			N1	N2	N3
Q.3	N1	Count	20	0	0
		Expected Count	9.5	5.5	5.1
		% within Ward Method	100.0%	0.0%	0.0%
		Adjusted Residual	5.9	−3.4	−3.3
		P (Zij)	0.000	0.001	0.001
	N2	Count	0	8	5
		Expected Count	6.1	3.5	3.3
		% within Ward Method	0.0%	61.5%	38.5%
		Adjusted Residual	−3.9	3.2	1.2
		P (Zij)	0.000	0.002	0.218
	N3	Count	6	7	9
		Expected Count	10.4	6.0	5.6
		% within Ward Method	27.3%	31.8%	40.9%
		Adjusted Residual	−2.4	0.6	2.1
		P (Zij)	0.015	0.537	0.032

**Table 9:** Post-hoc cellwise tests between the level of education and cluster of level of improvement of different attributes, after the implementation of new measures.

			Education		
			Lower & Intermediate	Upper Secondary School	Higher Education
Q.4.1	N1	Count	5	1	14
		Expected Count	5.5	2.2	12.4
		% within Ward Method	25.0%	5.0%	70.0%
		Adjusted Residual	−0.3	−1.1	0.9
		P (Zij)	0.7748	0.2880	0.3451
	N2	Count	4	5	4
		Expected Count	3.5	1.4	8.0
		% within Ward Method	30.8%	38.5%	30.8%
		Adjusted Residual	0.3	3.6	−2.6
		P (Zij)	0.7460	0.0003	0.0084
	N3	Count	6	0	16
		Expected Count	6.0	2.4	13.6
		% within Ward Method	27.3%	0.0%	72.7%
		Adjusted Residual	0.0	−2.1	1.4
		P (Zij)	1.0000	0.0341	0.1739

## 2 Experimental design, materials and methods

Data were obtained from experts highly involved in the design and implementation of conservation measures in Mozambique. The criteria used to select the experts were the following: (1) have worked or still work in Mozambique in conservation-related activities, irrespective of being Mozambican citizens; (2) have substantial knowledge about policies and laws that govern protected areas in Mozambique; and (3) know the current management state of the NNR including threats, compensation schemes and the role of all actors involved in conservation. The socio-demographic profile of surveyed experts is presented in Table 2. The questionnaire used to generate the dataset is presented in Appendix A. An online and self-administrated survey was presented to experts engaged in conservation in the NNR, in both Portuguese (Mozambican National Language) and English. The survey's main aim was to collect experts' perceptions and opinions on conservation-related issues, namely: (i) main practices threatening conservation in the NNR and those responsible for each practice; (ii) the reasons for local people's involvement with practices threatening conservation; (iii) effectiveness and limitations of current compensation measures to engage local people in conservation; and (iv) new measures that can be proposed to enhance conservation on the reserve. The survey also included a section on the socio-economic profile of respondents. The response rate was 68.76%, with two non-valid responses, that were dropped from the analysis.

The survey was coded in different rating scales depending on the question being analysed, according to the Excel spreadsheet and Table 3. Most of the questions were taken from the literature and brainstorming with a selected group of experts who have deep knowledge about conservation in NNR and other related conservation areas in the country. More detailed information about all the topics is available in Table 3 [1].

Respondents' ratings were first analysed through principal components for dimension reduction and subsequently to detect clusters structures. To understand whether there was any relationship between different views of respondents in all major themes, a crosstabulation between clusters was tested based on Fisher's Exact test and Asymptotic Person's Chi-Square [15,16]. When a significant relationship was detected, a post-hoc cellwise test (goodness-of-fit) was performed in order to find those attributes most significant for the association, and spell out the meaning of those relationships, based on the adjusted standardized residuals and adjusted alpha (α) [[17], [18], [19]]. The same technique was applied between clusters of major themes and socio-economic profile of respondents to understand whether their socio-economic background can also explain the points of views of respondents concerning major themes. Data from the post-hoc test is available in Table 4, Table 5, Table 6, Table 7, Table 8, Table 9 For more detailed information about the methodology see Mbanze et al. (2019) [1].
