# Supplementary material for: Dataset from 55 experts engaged in nature conservation in Mozambique
Source: Data Brief. 2020 Jan 3;28:105080. doi: 10.1016/j.dib.2019.105080 (PMC6965701; doi:10.1016/j.dib.2019.105080)
Supplement: Multimedia component 1 [file mmc1.docx]

**Appendix A and Supplementary material**.

The on-line survey can be downloaded from Google forms at:

<https://goo.gl/forms/ryuvRjl4xF5KDzjm1> (portuguese) and <https://goo.gl/forms/WUPfWwfDkW1kV0mq1> (english).

**Survey to the professionals and volunteers involved in the design and implementation of conservation measures in the Niassa National Reserve**

The Niassa National Reserve (NNR) is the largest conservation area in Mozambique. The Reserve was created for hunting propose in 1954 and later in 1997 was proclaimed a conservation area. It is one of the few remaining intact savannas in the world, and it’s also of global importance due to its biodiversity abundance, mostly endemic. Although efforts are being made to maintain the reserve, there are several problems that threaten biodiversity, mostly anthropogenic.

The present survey aims to collect the sensitivity of the professionals and volunteers involved in NNR conservation, in order to propose sustainable alternatives to improve the conservation. The survey has five sections and it is estimated that the average time to answer all questions ranges from 25 to 35 minutes. Questions marked with asterisk (*) must be answered. If you do not answer the question, the system will report an error alerts (an answer is required for this question), and you will not proceed to the next question before answering the previous one.

Your response to this survey is a valuable contribution to help us to propose consistent solutions to the current problems that threaten the degradation of the Reserve. We appreciate all attention and time you will spend, in order to help us to address this problem. There aren’t right or wrong answers to this questionnaire; all answers are important; we just want to know you point of view.

We will ensure total anonymity and confidentiality of your answers in the analysis and publication of all information will be collected.

If you find questions, doubts, comments or suggestions while you fill this questionnaire, you can contact: aires.banze@gmail.com or 26421@novasbe.pt, you can also send this questionnaire if you know people that will be interest to send to them.

We hope you will enjoy it too much

**SECTION I - PRACTICES INTERFERING WITH COSERVATION IN THE RESERVE**

1. **Could you please identify the degree of threat each of the existing problems in the Reserve represents to conservation?**

|  | **Very high** | **High** | **Moderate** | **Little** | **Very little** |
| --- | --- | --- | --- | --- | --- |
| Cut and burn agriculture | O | O | O | O | O |
| Commercial farming (e.g. tobacco, soy and cotton) | O | O | O | O | O |
| Sport hunting (Concessions / Hunting Blocks) | O | O | O | O | O |
| Poaching (e.g. ivory, bones, skin etc.) | O | O | O | O | O |
| Hunting for eat or sale meat locally (bushmeat) | O | O | O | O | O |
| Extraction of non-timber products (e.g. honey, fruits and roots) | O | O | O | O | O |
| Wood fuel (fire wood and charcoal) | O | O | O | O | O |
| Illegal logging | O | O | O | O | O |
| Fishing | O | O | O | O | O |
| Population growth in the reserve with increased pressure to the resources | O | O | O | O | O |
| Human and wildlife conflicts | O | O | O | O | O |
| Illegal gold and ruby mining | O | O | O | O | O |
| Projects and Infrastructures (roads, power poles and communication antennas) | O | O | O | O | O |

1. **Could you kindly add two more relevant problems and its respective degree of threats?**

Threat 1___________________________________________________________________

Threat 2___________________________________________________________________

1. **Indicate the main actor responsible for each problem described. In your answer, consider direct (action) or indirect responsibility (omission)**

|  | **Do** | **Go** | **ReAd** | **NoRe** | **LoPe** | **PrSc** | **TrAu** |
| --- | --- | --- | --- | --- | --- | --- | --- |
| Cut and burn agriculture | O | O | O | O | O | O | O |
| Commercial farming (e.g. tobacco, soy and cotton) | O | O | O | O | O | O | O |
| Sport hunting (Concessions / Hunting Blocks) | O | O | O | O | O | O | O |
| Poaching (e.g. ivory, bones, skin etc. | O | O | O | O | O | O | O |
| Hunting for eat or sale meat locally (bushmeat) | O | O | O | O | O | O | O |
| Extraction of non-timber products (e.g. honey, fruits and roots) | O | O | O | O | O | O | O |
| Wood fuel (fire wood and charcoal) | O | O | O | O | O | O | O |
| Illegal logging | O | O | O | O | O | O | O |
| Fishing | O | O | O | O | O | O | O |
| Population growth in the reserve with increased pressure to the resources | O | O | O | O | O | O | O |
| Human and wildlife conflicts | O | O | O | O | O | O | O |
| Illegal gold and ruby mining | O | O | O | O | O | O | O |
| Projects and Infrastructures (roads, power poles and communication antennas) | O | O | O | O | O | O | O |

**Where:** Do=Donors, ReAd=Reserve Administration, NoRe=Non-residents, LoPe=Local People, PrSc=Private,

Sector (PrSc) and TrAu=Traditional Authorities,

1. **There are several reasons for local people to be involved on practices that threaten conservation. Please, indicate your degree of agreement to each of the sentences given below**

|  | **Strongly Agree** | **Agree** | **Undecided** | **Disagree** | **Strongly Disagree** |
| --- | --- | --- | --- | --- | --- |
| Livelihood Insufficiency | O | O | O | O | O |
| Conservation does not bring any benefit, instead of restrictions | O | O | O | O | O |
| People don't know the importance of conservation | O | O | O | O | O |
| The local people are corrupted to corroborate with infractors | O | O | O | O | O |
| There are feeling of injustice in benefits sharing (especially 20% of the revenue) | O | O | O | O | O |
| Conservation only creates problems (e.g. human and wildlife conflict) | O | O | O | O | O |
| Conservation only benefits foreigners (e.g. visitors, government, NGOs, technicians or researchers) | O | O | O | O | O |
| The community are not strongly involved on the decision making and its implementation | O | O | O | O | O |
| Opposition to the restrictions imposed by conservation (e.g. hard rules and zoning) | O | O | O | O | O |
| Low education | O | O | O | O | O |
| Lack of infrastructure (e.g. hospitals, schools, transportation and communication) | O | O | O | O | O |

**SECTION II - EFFECTIVENESS AND LIMITATIONS OF THE COMPENSATION MEASURES**

*Different compensation measures are currently in place in the Niassa Reserve, to motivate local people in order to participate in the conservation activities*

1. **Put the measures listed below in order of importance to the local population, considering 1 = most important and 6 = least important**

|  | 1 | 2 | 3 | 4 | 5 |
| --- | --- | --- | --- | --- | --- |
| Jobs for the local population created under the conservation program, (e.g. Forest ranger position) | O | O | O | O | O |
| Hunting quotas allocated to communities | O | O | O | O | O |
| 20% income from the concessions which are delivered to the local people | O | O | O | O | O |
| Food allowances which are distributed to local people | O | O | O | O | O |
| Delivery of 50% of the revenue of the fines from these who detected the infraction in the reserve | O | O | O | O | O |
| Promotion and respect of culture and beliefs of local communities by government authorities and other actors in conservation (e.g. sacred places) | O | O | O | O | O |

1. **Some limitations have been referenced due to the way the compensation measures are being delivered. In this context, please indicate your agreement with the statements below**

|  | **Strongly Agree** | **Agree** | **Undecided** | **Disagree** | **Strongly Disagree** |
| --- | --- | --- | --- | --- | --- |
| Lack of transparency in the criteria to allocate the jobs position | O | O | O | O | O |
| The hunting quotas allocated to local people are not enough | O | O | O | O | O |
| The money allocated to the communities is not enough | O | O | O | O | O |
| Lack of monitoring and accountability in the use of 20% of concession revenues | O | O | O | O | O |
| In many cases, the detectors of the offenders do not receive the award | O | O | O | O | O |
| Weak training and advice to communities in how to use the compensation | O | O | O | O | O |
| Poor monitoring and evaluation of the results from the projects implemented to the benefit of communities | O | O | O | O | O |
| The above compensations are not enough to motivate the community | O | O | O | O | O |

**SECTION III**

*By improving existing measures and introducing new ones, it would be possible to encourage local people to participate in the conservation by adopting conservation friendly practices. Among these new measures, might be the introduction of individual or collectives’ incentives, in cash or in kind, (e.g. improved seeds, assistance in the production of certified products such honey, new techniques to keep away crop raids from farms and communities, as well as performance-based payments), which may lead to the improvement of the conservation performance. More than compensating local people, it’s a matter of motivating them to be more active in conservation. In the following questions, we would like to know your opinion regarding to the effectiveness of some of these new measures.*

1. **In your opinion, what will be the effectiveness of each of the measures described below in order to promoting the adoption of conservation-friendly practices by the household’s heads**

|  | **Very positive** | **Positive** | **Neutral** | **Negative** | **Very negative** |
| --- | --- | --- | --- | --- | --- |
| Create areas for cultivation of high-yield commercial crops (e.g. tobacco, corn, soybeans, cotton, etc.) to reduce pressure on land and obtain greater profits than others crop like cassava, maize etc. | O | O | O | O | O |
| Assist local people to the use environmentally-friendly cultivation practices (e.g. minimum cultivation, crop rotation, green manuring etc.) | O | O | O | O | O |
| Assist local people to produce alternative sources of animal proteins (e.g. chickens, pigs, poultry etc.) | O | O | O | O | O |
| Promoting certification of non-timber products (e.g. honey, fruit, medicinal plants etc.) in order to get higher market prices and encourage sustainable use of natural resources] | O | O | O | O | O |
| Training the communities for sustainable use of forest resources (timber, non-timber and fishing resources) | O | O | O | O | O |
| Involve local people in the management and decision-making on issues related to the reserve | O | O | O | O | O |
| Increase in the percentage of revenues charged to distribute to communities | O | O | O | O | O |
| Increased employment in conservation and recreation activities (e.g. tour guides, rangers, carpenters, hotels and restoration activities, etc.); | O | O | O | O | O |
| Attribution of collective conservation performance-based payments for local people | O | O | O | O | O |
| Provide education for local people (e.g. scholarships) | O | O | O | O | O |
| Improve services delivery for local people (e.g. health, education, roads etc.) | O | O | O | O | O |

1. **If you could only choose 4 of the measures mentioned above, what measures would you choose in order to improve the conservation status of the Niassa Reserve? You can mark only those you will be chosen**

| O | Create areas for cultivation of high-yield commercial crops (e.g. tobacco, corn, soybeans, cotton, etc.) to reduce pressure on land and obtain greater profits than others crop like cassava, maize etc. |
| --- | --- |
| O | Assist local people to the use environmentally-friendly cultivation practices (e.g. minimum cultivation, crop rotation, green manuring etc.) |
| O | Assist local people to produce alternative sources of animal proteins (e.g. chickens, pigs, poultry etc.) |
| O | Promoting certification of non-timber products (e.g. honey, fruit, medicinal plants etc.) in order to get higher market prices and encourage sustainable use of natural resources) |
| O | Training the communities for sustainable use of forest resources (timber, non-timber and fishing resources) |
| O | Involve local people in the management and decision-making on issues related to the reserve |
| O | Increase in the percentage of revenues charged to distribute to communities |
| O | Increased employment in conservation and recreation activities (e.g. tour guides, rangers, carpenters, hotels and restoration activities, etc.); |
| O | Attribution of collective conservation performance-based payments for local people |
| O | Provide education for local people (e.g. scholarships) |
| O | Improve services delivery for local people (e.g. health, education, roads etc.) |

1. **Could you justify the reason for the choice you made above?**

**Answer**______________________________________________________________________

_____________________________________________________________________________________

1. **If these 4 measures you selected in the previous question will be adopted in the reserve. What would be the percentage of improvement (on a scale of 0 to 100%), that you would expect to get from each conservation values?**

|  | **0%** | **1-25%** | **26-50%** | **51-75%** | **76-100%** |
| --- | --- | --- | --- | --- | --- |
| Increases of the biodiversity in general | O | O | O | O | O |
| Increases of forest cover | O | O | O | O | O |
| Increase of large carnivores and herbivores (e.g. the big fives); | O | O | O | O | O |
| Increment of fish stocks | O | O | O | O | O |
| Increase of large aquatic animals (e.g. crocodiles and hippos) | O | O | O | O | O |
| Reduction of degraded area due to cut and burn agriculture | O | O | O | O | O |
| Reduction of degraded area due to extraction of non-timber products | O | O | O | O | O |
| Reduction of local people engaged in illegal activities | O | O | O | O | O |
| Reduction of unsustainable trophy hunting (sport hunting)] | O | O | O | O | O |
| Reduction of illegal hunting for eat and sale the meat (bushmeat) | O | O | O | O | O |

1. **In addition to the conservation values above, what would be the improvement that you would expect to see in relation to these other attributes below?**

|  | **Very high** | **High** | **Moderate** | **Low** | **Null** |
| --- | --- | --- | --- | --- | --- |
| Knowledge of local communities regarding the importance of NNR conservation | O | O | O | O | O |
| Motivation of local people to participate in conservation activities | O | O | O | O | O |
| Disclosure of offenders | O | O | O | O | O |
| Mutual respect and trustiness among the different conservation actors | O | O | O | O | O |
| Increase of local people employed in the reserve | O | O | O | O | O |
| Reduction of human and wildlife conflicts | O | O | O | O | O |
| Reduction of frequency and forest fires intensity | O | O | O | O | O |

**SECTION IV - HOW TO CONCILIATE CONSERVATION, DEVELOPMENT AND LOCAL PEOPLE LIFESTYLE**

*Currently, most of the funding to support conservation activities in the reserve, are from international donors and hunting concessions.*

1. **Select at least four main sources of funding that can be explored to ensure the future sustainability of conservation activities in the reserve.**

| O | Donors |
| --- | --- |
| O | Government authorities |
| O | From tourism and ecotourism in general |
| O | Carbon credits and other related activities |
| O | Revenues from what is apprehended from illegal activities |
| O | Hunting concessions |

1. **Can you suggest at least two other sources of funding that can be explored**

One______________________________________________________________________________

Two______________________________________________________________________________

1. **Below are describe some factors that we believe you've pondered to select the four sources of funding. Could you please put order of importance these factors? In you answer, considers 1 = most important and 7 = least important**

|  | **1** | **2** | **3** | **4** | **5** | **6** | **7** |
| --- | --- | --- | --- | --- | --- | --- | --- |
| Job generation | O | O | O | O | O | O | O |
| Potential to generate revenue | O | O | O | O | O | O | O |
| Attracting of investments and its viability | O | O | O | O | O | O | O |
| Sustainable conservation activities | O | O | O | O | O | O | O |
| Empowering local communities; | O | O | O | O | O | O | O |
| Potential to improve local people intellectual and financial capacities | O | O | O | O | O | O | O |
| Potential to reduce external influence in the conservation policies. | O | O | O | O | O | O | O |

1. **Can you please mention another important factor that you took in consideration, which was not described above?**

____________________________________________________________________________________________________________________________________________________________

**SECTION V - SOCIO-ECONOMIC PROFILE OF RESPONDENTS**

1. **Gender**

| O | Female |
| --- | --- |
| O | Male |
| O | Prefer not to say |

1. **Your major field**

| O | Anthropology |
| --- | --- |
| O | Biology |
| O | Social Sciences |
| O | Political Sciences |
| O | Conservation of Natural Resources |
| O | Rural development |
| O | Ecology |
| O | Economics |
| O | Agricultural Engineering |
| O | Forest Engineering |
| O | Environmental Engineering |
| O | Rural Extension |
| O | Geography |
| O | Medicine |
| O | Others |

1. **Degree of Education**

| O | Elementary Education |
| --- | --- |
| O | Lower Secondary School |
| O | Lower Professional Education |
| O | Intermediate Professional Education |
| O | Upper Secondary Education |
| O | BSc/ Graduation Degree |
| O | Master’s degree (M.Sc.) |
| O | PhD |
| O | Post-doctoral |
| O | Other (Indicate please) |

1. **How many times have you been in the Niassa Reserve?**

Never__; 1___; 2____; 3 – 5 ____; > 5 _____

1. **For how long time you stayed in the reserve? (sum of all trips) and what was the main objective of your trip to the reserve**

| O | Any time | O | Tourism |
| --- | --- | --- | --- |
| O | Less than a month | O | Research |
| O | 1 – 4 Months | O | Visit friend family |
| O | 5 – 8 Months | O | Just passing through |
| O | 8 – 12 Months | O | Working |
| O | > 12 Months | O | Others |

1. **Years of experience in conservation (if applicable)**

Any___; 1____; 2 – 5____; 6 – 10____; >10_____

1. **Your current institution** ___________________________________________________________
2. **Position** _______________________________________________________________________
3. **Other institutions where you worked before**__________________________________________

*If you find it relevant, provide email contact from two persons who would be relevant to send this questionnaire. You can also send personally. Don't forget to provide full name and the respective institutions where the people work*

*Thank you for your time*
